# Supplementary material for: The association between childhood maltreatment and empathic perspective taking is moderated by the 5-HTT linked polymorphic region: Another example of “differential susceptibility”
Source: PLoS One. 2019 Dec 19;14(12):e0226737. doi: 10.1371/journal.pone.0226737 (PMC6922468; doi:10.1371/journal.pone.0226737)
Supplement: S1 Table — (PDF) [file pone.0226737.s001.pdf]

S1 Table. Data from genotyping, CTQ and IRI questionnaires for each participant.

Participant No.	5-HTTLPR genotype	rs25531 genotype	CTQ total score	IRI perspective taking
1	LS	La/Sa	65	19
2	LL	Lg/La		16
3	LS	Lg/Sa		12
4	LL	La/La	76	11
5	LL	La/La	67	19
6	LS	La/Sa	74	10
7	SS	Sa/Sa	82	
8	LS	La/Sa	33	21
9	LS	La/Sg	100	11
10	SS	Sa/Sa		14
11	SS	Sa/Sa		16
12	LL	La/La		5
13	LS	La/Sa	103	3
14	LL	Lg/La	84	16
15	LL	Lg/La	59	15
16	LL	Lg/La	60	13
17	LS	La/Sa	61	13
18	LL	La/La		
19	SS	Sa/Sa	64	20
20	LL	La/La		9
21	LS	Lg/Sa	44	0
22	LS	La/Sa		14
23	LL	La/La		1
24	LS	La/Sa	59	
25	LS	La/Sa		14
26	LL	La/La		11
27	LS	La/Sa	61	17
28	LL	La/La		12
29	LS	La/Sa	34	13
30	LS	La/Sa		17
31	LL	La/La		9
32	LL	La/La	54	19
33	SS	Sa/Sa		18
34	LS	La/Sa		17,5
35	LL	La/La	41	7
36	SS	Sa/Sa		21
37	LL	La/La		
38	SS	Sa/Sa		14
39	LS	La/Sa	85	22
40	LS	Lg/Sa		9
41	LL	La/La	75	4
42	LL	La/La	92	18
43	LS	La/Sa	76	3
44	LL	Lg/La	39	9
45	LS	La/Sa		
46	LS	La/Sa	99	19
47	LL	Lg/La	86	11
48	LL	La/La		10
49	LL	La/La		14
50	SS	Sa/Sa	87	18

51	SS	Sa/Sa		18
52	LL	La/La	65	8
53	SS	Sa/Sa	35	17
54	LS	La/Sa	53	16
55	LL	Lg/La	46	22
56	LS	La/Sa		16,5
57	LL	La/La	78	16
58	LL	La/La	38	10
59	LL	La/La	82	13
60	LS	La/Sa	73	18
61	LS	La/Sa	51,5	16
62	LS	La/Sa	62	17
63	LS	La/Sa	62	24
64	LL	La/La	60	6
65	SS	Sa/Sa	39	28
66	LS	La/Sa		
67	LL	La/La	73	18
68	LS	La/Sa	33	10
69	LS	La/Sa	60	13
70	LL	La/La	67	28
71	LS	Lg/Sa	35	13
72	LS	La/Sa	73	8
73	LS	La/Sa	60	20
74	LL	La/La	48	12
75	LL	Lg/La	42	12
76	LS	La/Sa		
77	LS	La/Sa	91	
78	LL	La/La	36	8
79	LS	La/Sa		
80	LS	La/Sa	27	8
81	LL	La/La	63	13
82	LS	La/Sa	107	15
83	LL	La/La		20
84	LS	Lg/Sa	92	9
85	LS	La/Sa	34	16
86	LL	Lg/La	70	13
87	LS	La/Sa		
88	LS	La/Sa	79	6
89	LS	La/Sa	60	
90	LL	La/La	41	
91	LL	Lg/La	85	14
92	LL	La/La		
93	LL	La/La	59	12
94	SS	Sa/Sa	73	17
95	LS	La/Sa	44	9
96	LS	La/Sa	44	23
97	LS	La/Sa	79	8
98	LS	La/Sa	33	10
99	LL	La/La	66	20
100	SS	Sa/Sa		
101	LS	Lg/Sa	83	9
102	LL	La/La	101	20
103	LL	Lg/La	71	15

104	SS	Sa/Sa	63	16
105	LS	La/Sa	60	9
106	LS	La/Sa	72	11
107	LS	Lg/Sa	87	4
108	LS	Lg/Sa	45	
109	LL	Lg/La	73	8
110	SS	Sa/Sa	101	12
111	LS	La/Sa	72	2
112	LS	Lg/Sa	55	6
113	LL	La/La		
114	SS	Sa/Sa	47	18
115	LL	La/La	64	14
116	LL	La/La	80	19
117	LS	La/Sa	35	12,5
118	LL	La/La	84	33
119	LS	La/Sa	64	15
120	LL	La/La	39	19
121	LL	Lg/La	56	
122	LS	La/Sa	78	16
123	LL	La/La	72	8
124	LS	La/Sa	71	16
125	LS	La/Sa	95	8
126	LL	La/La	43	12
127	SS	Sa/Sa	80	14
128	LL	Lg/La	52	16
129	LL	La/La		
130	SS	Sa/Sa	53	17
131	LL	La/La	49	
132	SS	Sa/Sa	62	17
133	LL	La/La	69	16
134	LL	La/La	71	7
135	LS	La/Sa	65	19
136	SS	Sa/Sa	37	
137	LS	Lg/Sa	59	11
138	LS	La/Sa	37	
139	LL	Lg/La	33	20
140	LS	La/Sa	44	
141	LS	La/Sa	83	19
142	LL	La/La	97	14
143	LS	La/Sa		12
144	LS	Lg/Sa		19
145	LL	Lg/La		16
146	LL	La/La	28	11
147	LS	Lg/La	27	23
148	LS	La/Sa		17
149	LL	La/La		19
150	LS	La/Sa	27	25
151	LS	La/Sa		24
152	SS	Sa/Sa		14
153	LL			17
154	LS	La/Sa		23
155	LL	Lg/La		
156	LL	La/La	30	19

157	LS	La/Sa		24
158	LL	La/La	29	26
159	LL	La/La		21
160	LS	La/Sa		22
161	LS	La/Sa		25
162	LS	La/Sa		27
163	LL	La/La		13
164	LL	La/La	27	18
165	LS	La/Sa		24
166	LL	La/La	26	22
167	SS	Sa/Sa		17
168	LS	La/Sa		19
169	SS	Sa/Sa	26	23
170	SS	Sa/Sa		17
171	LL	La/La		21
172	SS	Sa/Sa		22
173	LL	La/La		13
174	LS	La/Sa		
175	LS	La/Sa		14
176	SS	Sa/Sa	29	15
177	LS	La/Sa		20
178	LS	La/Sa	31	19
179	LS	La/Sa	32	20
180	LS	La/Sa	32	16
181	LS	La/Sa	37	18
182	LS	La/Sa	27	21
183	LS	La/Sa	40	19
184	LS	La/Sa	31	13
185	LL	La/La	28	14
186	LS	La/Sa	27	19
187	LS	La/Sa	29	20
188	LS	La/Sa	33	16
189	LL	La/La	27	14
190	LS	La/Sa	26	11
191	SS	Sa/Sa	37	18
192	LL	La/La	45	9
193	LL	Lg/La	43	18
194	LS	Lg/Sa	28	16
195	LL	La/La	25	13
196	LS	Lg/Sa	26	20
197	LS	La/Sa	29	23
198	SS	Sa/Sa	35	25
199	LS	La/Sa	29	15
200	LS	Lg/Sa	35	21
201	SS	Sa/Sa	26	24
202	SS	Sa/Sa		15,5
203	LL	La/La	32	22
204	LL	La/La	39	22
205	LL	La/La	31	24
206	LL	La/La	25	18
207	LS	La/Sa	31	25
208	LL	La/La	28	16
209	LS	La/Sa	62	24

210	LL	Lg/La	29	16
211	LL	La/La		
212	LS	La/Sa	36	19
213	LL	La/La	66	20
214	LL	La/La	63	26
215	LL	La/La	33	19
216	LS	La/Sa	36	25
217	LL	La/La	66	27
218	LL	Lg/Lg	63	21
219	LS	La/Sa	33	27
220	LL	La/La	42	21
221	LS	La/Sa	25	15
222	LL	Lg/La	32	21
223	LS	La/Sa	31	17
224	LS	La/Sa	26	16
225	LS	La/Sa	45	5
226	LS	La/Sa	28	20
227	LL	Lg/La	27	21
228	LL	Lg/La	46	17
229	LL	La/La	32	17
230	LL	La/La	40	21
231	LL	Lg/La	58	21
232	SS	Sa/Sa	35	23
233	SS	Sa/Sa	36	15
234	LS	La/Sa	30	16
235	LS	Lg/Sa	88	16
236	LL	Lg/La	55	24
237	SS	Sa/Sa	28	22
238	SS	Sa/Sa	28	21
239	LS	La/Sa	34	21
240	SS	Sa/Sa	38	10
241	LL	Lg/La	25	16
242	LS	La/Sa	34	13
243	SS	Sa/Sa	33	19
244	SS	Sa/Sa	25	22
245	SS	Sa/Sa	29	10
246	LS	La/Sa	30	21
247	SS	Sa/Sa	30	20
248	SS	Sa/Sa	32	25
249	LS	La/Sa	44	23
250	LS	La/Sa	25	18
251	LS	La/Sa	26	21
252	LL	Lg/La	35	25
253	SS	Sa/Sa	45	19
254	LS	La/Sa	43	18
255	LL	La/La	29	21
256	LS	La/Sa	25	20
257	SS	Sa/Sa	28	22
258	LS	La/Sa	26	15
259	LS	Lg/Sa	26	22
260	LS	La/Sa	29	19
261	LS	La/Sa	32	25
262	LS	La/Sa	26	23

263	LS	La/Sa	25	22
264	SS	Sa/Sa	25	24
265	LS	La/Sa	25	16
266	LS	La/Sa	25	18
267	LS	La/Sa	29	25
268	LS	La/Sa	28	19
269	LL	La/La	38	14
270	LS	La/Sa	29	12
271	LS	La/Sa	29	26
272	SS	Sa/Sa	26	17
273	LL	La/La	32	24
274	LS	Lg/Sa	29	25
275	LS	La/Sa	32	15
276	SS	Sa/Sa	34	24
277	LL	La/La		
278	LS	La/Sa	26	22
279	LS	La/Sa	26	18
