# Supplementary material for: The association between childhood maltreatment and empathic perspective taking is moderated by the 5-HTT linked polymorphic region: Another example of “differential susceptibility”
Source: PLoS One. 2019 Dec 19;14(12):e0226737. doi: 10.1371/journal.pone.0226737 (PMC6922468; doi:10.1371/journal.pone.0226737)
Supplement: Attachment — Submitted filename: Reponse to Reviewers.docx [file pone.0226737.s002.docx]

**Revision 1**

PONE-D-19-14573
The association between childhood maltreatment and empathic perspective taking is moderated by the 5-HTT linked polymorphic region. Another example of “differential susceptibility”

**Journal Requirements:**

1. When submitting your revision, we need you to address these additional requirements.

Please ensure that your manuscript meets PLOS ONE's style requirements, including those for file naming. The PLOS ONE style templates can be found at
<http://www.journals.plos.org/plosone/s/file?id=wjVg/PLOSOne_formatting_sample_main_body.pdf> and <http://www.journals.plos.org/plosone/s/file?id=ba62/PLOSOne_formatting_sample_title_authors_affiliations.pdf>

2. Please include captions for your Supporting Information files at the end of your manuscript, and update any in-text citations to match accordingly. Please see our Supporting Information guidelines for more information: <http://journals.plos.org/plosone/s/supporting-information>.

3. We note that you have indicated that data from this study are available upon request. PLOS only allows data to be available upon request if there are legal or ethical restrictions on sharing data publicly. For information on unacceptable data access restrictions, please see <http://journals.plos.org/plosone/s/data-availability#loc-unacceptable-data-access-restrictions>.

In your revised cover letter, please address the following prompts:

a) If there are ethical or legal restrictions on sharing a de-identified data set, please explain them in detail (e.g., data contain potentially identifying or sensitive patient information) and who has imposed them (e.g., an ethics committee). Please also provide contact information for a data access committee, ethics committee, or other institutional body to which data requests may be sent.

b) If there are no restrictions, please upload the minimal anonymized data set necessary to replicate your study findings as either Supporting Information files or to a stable, public repository and provide us with the relevant URLs, DOIs, or accession numbers. Please see <http://www.bmj.com/content/340/bmj.c181.long> for guidelines on how to de-identify and prepare clinical data for publication. For a list of acceptable repositories, please see <http://journals.plos.org/plosone/s/data-availability#loc-recommended-repositories>.

We will update your Data Availability statement on your behalf to reflect the information you provide.

🡪 Response: We revised the manuscript with regards to the journal requirements. We also add the study findings as supporting information.

**Reviewer #1**:

This study focuses on the effect of 5-HTT linked polymorphic region in the association between childhood maltreatment and empathic perspective taking. In 279 female patients with BPD or healthy participants, childhood maltreatment and empathic abilities were measures using standardized questionnaires and genotypes in 5-HTT linked polymorphic region were determined and further categorized by different levles of transcriptional activities. Moderator analysis revealed that S-allele carriers and corresponding lower transcriptional activities showed significant response to childhood mental environmental impact in empathic perspective taking.

Previous studies mainly investigated the association negative environmental impact in empathic perspective taking while this study demonstrated the bidirectional empathic perspective taking outcome from childhood treatment.


Major:
1. How was the effect of recent major life events in adulthood evaluated for the 279 participants, which may serve as controls in this study.

Response: Unfortunately, we cannot exclude or control for effects of recent/present life events, because we did not assess traumatic experiences during adulthood. Since the reviewer raised an important point with this concern, we add this point to the limitation section. This part reads as follows: “*Forth, since we assessed only adversity during childhood we were unable to exclude confounding effects of recent negative life events, which may also have impact on present social behavior. Thus, future studies may investigate childhood, as well as recent adversity, in order to define the concrete contribution of these factors on social behavior and the development of psychopathology.*”

2. Moderator analysis of CTQ total score, perspective taking and the BPD/ healthy participants.

Response: We agree with the reviewer that is important to show that the associations found were not already coming up because of the BPD and HC groups. Therefore, we added another moderation analysis with Group (BPD/HC) as the moderator. We added the following part to the methods: “*Another moderation analyses was performed for the SS, SL and LL Genotypes and for Group (BPD vs HC) as the moderator instead of Genotype. “*

The following part was added to the results section: “*We further aimed to examine whether the association of childhood trauma with perspective talking was related to diagnosis. Therefore, we performed the same moderation analysis but tested the factor group (BPD and HC) as the moderator. The model was also significant (F(5, 199) = 11.71, p < 0.001, R² = 0.2272), but the interaction of CTQ by group was not (Interaction b = 0.0437, t(199) = 0.81 , p = 0.419), suggesting that a diagnosis of BPD was not the sole factor impacting on the association of childhood trauma with empathic perspective taking “*

We also extended the limitation section in the discussion: “However, since our main interest pertained to the influence of childhood adversity on trait empathy and its moderation by genotype, we were much less concerned with the presence or absence of specific effects of a diagnosis of BPD. *In support of this idea, the moderation analysis with the moderator group (BPD/HC) did not show a specific effect of BPD on the association of childhood trauma with perspective taking.”*

Minor:
1. How does the CTQ questionnaire define age range of childhood?

Response: The age range is not clearly defined by the questionnaire. The instruction on the questionnaire asks the participants to refer to their childhood and adolescence. This information was added to the methods: “Participants were asked to rate the occurrence of maltreatment on a 5-point Likert scale (1 = never, 5 = very often) *pertaining to their childhood and youth.*”

2. Moderator analysis on three genotypes (Line 264-268) can be moved after moderator analysis on SS+SL vs LL (after line 244).

Response: We agree with the reviewer and change the manuscript accordingly

3. Typo: genotype should be genotypes in line 264.

Response: Right, thanks for the hint!

4. In Table 3 and regarding description, the total n in the SS+SL group was stated as 176, however, only 175 samples can be identified.

Response: We verified the data shown in Table 3 several times, but could not find a mistake.

5. In Table 3, LGSG group or SGSG group are also not presented in patients or healthy participants. Either remove the SASG group from the table or add these groups.

Response: We agree with the reviewer and decided to remove the SASG group.

6. In the discussion (lines 287-291), the author’s statement regarding the benefit of emotional warmth and availability during childhood is confusing.

Response: we revised the part in order to make it more clearly: “As noted by Belsky et al. [63], the mere absence of maltreatment is not equivalent to high-quality parenting. *When applying this idea to the present findings, it can tentatively be hypothesized that* emotional warmth and availability during early development may lead to even better-than-average perspective-taking abilities (in this case, the slopes of the regression lines shown in Fig 1 may diverge to a greater extent if extrapolated to the left)*.*”

**Reviewer #2:**

In this manuscript, authors investigate the role of 5-HTTLPR on the effects of child maltreatment on empathy. In a sample of 279 women (142 with BPD and 137 healthy controls), they studied the effects of 5-HTTLPR genotype on empathic perspective taking. The manuscript is well-written, but not rigorous in the study design and methods. Further, the conclusions made are premature, given the lack of replication and the numerous weaknesses identified.

1. There is a lack of power due to the small sample size of their cohort, particularly for the interaction analysis. Authors should include a power analysis to justify the sample size used in the study.

Response: Following up on the reviewer’s concern, we added a power analysis for the interaction effect: “*We conducted a power analysis for the interaction, i.e. the differences between slopes for the moderation model with the genotypes SS+SL and LL. Therefore, we used G*Power Version 3.1.9.2. (56). For the calculation of the achieved power with the present sample for the interaction effects we applied the option “t-test-linear bivariate Regression, two groups, difference between slopes”, which showed, that we reached a statistical power of α = 0.749 with a sample size of 205 participants.*”

We also extended the discussion accordingly: “*In any event, even though the statistical power for detecting significant interaction effects was sufficiently large with α = 0.75, it, it is warranted to replicate the study in an independent, and preferably larger, sample.”*

In addition, we would like to mention that other recent work utilized comparable sample sizes (for example Nardi et al., 2013; Owens et al., 2012; Wagner et al., 2009, for review on 5-HTTLPR and psychopathology see Kenna et al., 2012)

2. All subjects should have trauma information, including controls, since one of the main analysis performed is an interaction analysis.

Response: The moderation analyses were conducted for the whole sample, e.g. controls and BPD (except missing data). Participants of both groups received the CTQ (see also Table 2)

3. There is no information about race. This should be also accounted for in the analysis.

Response: The reviewer is absolutely right with expressing concern regarding the ethnical background in a genetic study. We added the information to the material and methods – participant section. “*Regarding the ethnical background, 95.2 % were Caucasians, 4.4 % originated from the Middle East (mainly of Turkish origin) and 0.4 % from the Far East (Vietnamese).*”

4. Depression is highly prevalent among BPD. Analysis should account for depression as a covariate.

Response: We agree with the reviewer and added moderation analyses, which include the covariate BDI score.

The methods were extended as following: “*In addition, we used the Beck’s Depression Inventory to assess the self-rated level of depressivity [54* […] “*In order to examine the impact of depressivity, we calculated additional moderation analyses with the additional covariate “BDI score” for the moderators “SS+SL vs. LL”, “SS, SL and LL” and transcriptional activity.*”

We extended the results accordingly (see also Table 2): “Patients differed in age and IQ from control participants and reported more severe experiences of childhood maltreatment *as well as more depressive symptoms*.” […] “*Since depressivity is highly prevalent among individuals suffering from BPD, we repeated the moderation analyses and included also the BDI score as a covariate into the calculation. Here, the same results were obtained as in the previous analyses without the covariate BDI (moderator = SS+SL vs. LL: the model (F(6, 196) = 9.20, p < 0.001, R² = 0.2198) and the interaction (b = -0.0857, t(196) = -2.38 , p = 0.018) were significant; moderator = SS vs.SL vs. LL: the model was significant (F(6, 196) = 8.81, p < 0.001, R² = 0.2124), the interaction showed only a tendency (b = -0.0479, t(196) = -1.84 , p = 0.067); moderator TA group: the model (F(6, 196) = 9.43, p < 0.001, R² = 0.2239) and interaction (b = -0.0649, t(196) = -2.63 , p = 0.009) were significant).*

We also discussed the association with depressivity more extensively, as raised by Reviewer (Comment 7) and referred to our results concerning depression: “[…] *In our study, the inclusion of depression as a covariate did not affect the interaction, which suggests that the effect on perspective taking was not due to symptoms of depression. This further implies that our study does also not support the link between 5-HTTLPR, stress and depression*.[…]”

5. No information about healthy controls group is provided. What is the prevalence, if any, of disorders and medication listed Table 1 for BPD group.

Response: We agree with the reviewer that no information was given. We added the following sentence to the methods: “*The control participants were free of medication and psychiatric disorders.”*

6. No efforts for replication were made. Given the sample size, it is critical to conduct a replication analysis in an independent cohort.

Response: We absolutely agree with the reviewer that a replication in a larger sample is required and included this point in the discussion.

Discussion: “*In any way, since the power for detecting significant interaction was 0.749, it is warranted to replicate the study in an independent, and preferably larger, sample.*

7. In the largest meta-analysis studying the interaction between stress and 5-HTTLPR genotype in the context of depression, the Culverhouse et al found no association. Authors should discuss why studying 5-HTTLPR genotype is still relevant.

Response: We are grateful for the advice to extend the discussion to the depression debate and added the following section to the discussion: “*With regard to depression, the exact role of the 5-HTTLPR in the development of depression is unclear. This was shown by recent meta-analyses, which reported on the one side an association of stressful live events and depression in SS-homozygotes or S-carrying heterozygotes [42, 43], and on the other side other studies found no significant association [31, 44]. Culverhouse and colleagues found a significant main effect of sex and life stressor (high risk factor for depression), but they did not found an effect of genotype on the association of stress and depression, even if they included only studies with large sample sizes [44]. The authors concluded that if any interaction would exist, it would not be a generalizable effect, only detectable in limited situations and of modest effect size. In our study, the inclusion of depression as a covariate did not affect the interaction, which suggests that the effect on perspective taking was not due to symptoms of depression. This further implies that our study does also not support the link between 5-HTTLPR, stress and depression. Eventually, the 5-HTTLPR, together with stress, may induce stress and emotion processing impairments (as described above), which lead only in a subset of individuals to the development of depression. This subset may bear additional risks, which are currently not in the focus of interaction studies. One potential factor could be the transcriptional activity of the serotonin transporter gene, which could be altered by epigenetic processes or other SNPs, as for example the rs25531 [33, 34, 67].*
