# Supplementary material for: The association between childhood maltreatment and empathic perspective taking is moderated by the 5-HTT linked polymorphic region: Another example of “differential susceptibility”
Source: PLoS One. 2019 Dec 19;14(12):e0226737. doi: 10.1371/journal.pone.0226737 (PMC6922468; doi:10.1371/journal.pone.0226737)
Supplement: Attachment — Submitted filename: Reponse to Reviewers.docx [file pone.0226737.s003.docx]

**Revision 2**

PONE-D-19-14573
The association between childhood maltreatment and empathic perspective taking is moderated by the 5-HTT linked polymorphic region. Another example of “differential susceptibility”

**Reviewer #1**:

Considering the study focuses on childhood adversity instead of lifetime adversity, better definition in age and evidence of adversity only in childhood are critical in the study design

Response: We are grateful for the reviewer’s advice. Accordingly, we explicitly make the distinction between childhood trauma and other adverse life events in the discussion. In addition, we now point out, throughout the manuscript, that the present study deals with early adversity.

**Reviewer #2:**

Major comments:
* There were three moderation analyses with the same outcome and predictor variables by the different variables: 1) S allele carriers vs non-carriers; 2) transcriptional activity (L/A genotype vs others); and 3) the BPD status. Although all the summary statistics were written down in the result section, it would be much better for the audience if they summarize the statistics into a table.

Response: We are thankful for the comment and created table 5, which shows the moderator variables, covariates and statistics of the moderation analyses conducted (page 15). The table was a great advice, since results are more comprehensive and accessible for the reader. We therefore deleted the results of analyses with BDI as an additional covariate, since it can be found in the table.

**Table 5. Summary of moderation analyses performed for the predictor variable “CTQ” (total score) and the outcome variable “perspective taking”. The table shows the moderator variables and covariates used and the respective model statistics and interactions between the predictor and moderator variables. The last two columns show tests for differential susceptibility, i.e. differences between slopes and differences from zero.**

| **Moderator** | **Covariate** | **Model** | **Interaction moderator*CTQ** | **Differences of slopes from 0** | **Differences between slopes** |
| --- | --- | --- | --- | --- | --- |
| SS+SL vs. LL | age, IQ | F(5, 199) = 6.48, *p* < 0.001, *R²* = 0.1401 | *b* = -0.0810, *t*(199) = -2.16 , *p* = 0.032 | SS+SL *b* = -0.109, SE = 0.023, *p* < 0.001 | *t* = 3.08, *p* = 0.002 |
|  |  |  |  | LL: b = -0.028,  SE = 0.031, p = 0.378 | |
|  | age, IQ, BDI | (*F*(6, 196) = 9.20, *p* < 0.001, *R²* = 0.2198 | *b* = -0.0857, *t*(196) = -2.38 , *p* = 0.018 |  |  |
| SS vs. SL vs. LL | age, IQ | *F*(5, 199) = 6.77, *p* < 0.001, *R*² = 0.1374 | *b* = -0.0497, *t*(199) = -1.84 , *p* = 0.067 |  |  |
|  | age, IQ, BDI |  | *b* = -0.0479, *t*(196) = -1.84 , *p* = 0.067 |  |  |
| TA groups: low/low, high/low, high/high | age, IQ | *F*(5, 199) = 6.77, *p* < 0.001, *R*² = 0.1453 | *b* = -0.0624, *t*(199) = -2.44 , *p* = 0.015 | high/high: *b* = -0.0378, SE = 0.0254, *p* = 0.1389 | high/high vs. low/low: *t* = 3.30, *p* = 0.001 |
|  |  |  |  | high/low: b = -0.0808, SE = 0.0189, p < 0.001; | high/low vs. low/low: t = 2.00, p = 0.047 |
|  |  |  |  | low/low: b = -0.1237, SE = 0.0262, p < 0.001 | high/high vs. high/low group t = 2.01, p = 0.046 |
|  | age, IQ, BDI | *F*(6, 196) = 9.43, *p* < 0.001, *R*² = 0.2239 | *b* = -0.0649, *t*(196) = -2.63 , *p* = 0.009 |  |  |
|  |  |  |  |  |  |
| BPD vs. HC | age, IQ | *F*(5, 199) = 11.71, *p* < 0.001, *R*² = 0.2272 | *b* = 0.0437, *t*(199) = 0.81 , *p* = 0.419 |  |  |

* The author did power calculation using G*Power, but they did not explain about how they computed in detail at all. They should explicitly explain the parameter values (e.g. expected effect size and significance threshold) in the method section.

Response: We extended the section on the power calculation as follows: Page 8 line 161-165: “We conducted a power analysis for interaction effects, i.e. the differences between slopes for the moderation model with the genotypes SS+SL and LL, using G*Power, Version 3.1.9.2. [59]. *Power calculation for the current sample of 205 participants was determined by the following model: t-test-linear bivariate regression, two groups, difference between slopes, with α set at 0.05. Standard deviation of the residuals, the sample size and the difference between the slopes were also considered. Accordingly, the statistical power coefficient was 0.75.* “

Minor comments:
* There are many different statistical tests in the manuscript. I think the main analysis is the moderation analysis. So, it would be nice if author better shows the three main moderation analysis models at once in the method section. It could be a formula format or a table. This will really improve understanding of this study.

Response: The authors completely agree with the reviewer that the statistical methods section was very long and difficult to follow. We therefore added another table showing the moderation analyses (page 10)

**Table 2. Summary of moderation analyses conducted. The predictor and outcome variables remained constant across calculations whereas the moderator and control variables were exchanged.**

| **Predictor** |  | **Outcome** | **Moderator** | **Covariates** |
| --- | --- | --- | --- | --- |
| CTQ  total score |  | Perspective  taking | SS+SL vs. LL | age, IQ |
|  |  |  |  | age, IQ, BDI |
|  |  |  | SS vs. SL vs. LL | age, IQ |
|  |  |  |  | age, IQ, BDI |
|  |  |  | TA groups: low/low, high/low, high/high | age, IQ |
|  |  |  |  | age, IQ, BDI |
|  |  |  | BPD vs. HC | age, IQ |

* I think the full name of MANOCOVA should be defined in the page 8 (line number: 165).

Response: We changed this sentence accordingly (now page 9 line 179): “In order to investigate the effect of the genotype, we calculated a *multivariate analysis of covariance* (MANCOVA) with the covariates IQ and age and the between-subject factor genotype (SS+SL vs. LL) and the independent variables were the IRI scores.”

* I think you meant the moderator (M: low/low, high/low and high/high), rather than (M: low/low, high/low and low/low), in the page 13 (line number: 253).

Response: Correct, this was an error, thank you for mentioning!

* I am not sure why the subtitle, "Genotyping" was written in Italic font in the page 7 (line number 145).

Response: Sorry, this shouldn’t be italic and was corrected.

* Grammar error. "by calculation the correlation" in the page 8 (line number 176).

Response: Thank you for the hint, we improved the sentence to “by *calculating* the correlation of…”

* What does "X2(2)" mean in the page 10 (line number: 204)?

Response: The (2) stands for the df of the Chi square test, we changed it to: “(*X*² = 2.84; *p* = 0.241; df = 2; Table 4)”

* Author wrote why they divided the sample into S carriers vs. LL carriers in the result section in the page 10 (line number: 205-206). I think this should go into the method section. Also, it would be nice if author briefly explain about the rationale behind this stratification by S allele.

Response: We transferred the section into the methods section and added the following part explaining the rationale (page 8 line 166-172): “*In accordance with previous studies, we divided the sample into S-carriers (SS+SL pooled) and LL-carriers (e.g. [60-62]). This approach was justified, because previous studies reported no differences between SS and SL-carriers with regard to personality traits, suggesting a dominant-recessive type of association of the S-allele with personality (21).*

*Similarly, following previous research (e.g., [37]), subjects were further divided into groups according to the ”transcriptional activity“ (TA) of the rs25531, which is reported in detail in Table 4.* “

The results were changed to (page 11 line 223-226): “*The division into S or LL-carriers resulted in n = 103 LL-carriers and n = 176 S-carriers. Another group formation was based on the rs25531, which offers the opportunity to build “transcriptional activity“ (TA) groups, (see Table 4)*.”

* I found that something should go into the discussion section other than in the result. The sentence in the page 12 (line number: 231-232) is the interpretation about the result from the moderation test.

Response: The authors agree with the reviewer and deleted the interpretation in the results.

* It was not described how the moderation analysis for the three genotypes was conducted. Did you use the additive model? It should be described in the method section.

Response: The moderation analysis of the three genotypes can be found on page 9 line 194-196 and now in the table 2.

* It would be nice if the authors show the LD information between the S/L variations and rs25531. Also, the allele frequencies of these variations in general population could be relevant information, which may exist in the gnomAD (<https://gnomad.broadinstitute.org/>) database.

Response: We inserted the information into the introduction (page 3 line 69-71, page 4 line 73-76): “However, this polymorphic variation does not generally occur more frequently in clinical samples compared to the general population [30-32]. *The general population is heterozygous, whereas the LL-genotype is less common, and the SS-variant relatively rare, in part depending on ethnicity [33, 34].* In addition, controversy exists about the effect of rs25531, a SNP within the 5-HTTLPR repetitive element, with the A-variant of the L-allele being associated with greater transcriptional activity and thus more efficient serotonin turnover [35-37], *whereby a linkage disequilibrium between 5-HTTLPR and rs25531 has been described, with the rarer G-variant of rs25531 occurring more frequently together with the L-allele than with the S-allele [36, 38].*
